# The Association Between Cyclooxygenase-2 –1195G/A (rs689466) Gene Polymorphism and the Clinicopathology of Lung Cancer in the Japanese Population: A Case-Controlled Study

**DOI:** 10.3389/fgene.2022.796444

**Published:** 2022-04-05

**Authors:** Rong Sun, Ryosuke Tanino, Xuexia Tong, Minoru Isomura, Li-Jun Chen, Takamasa Hotta, Tamio Okimoto, Megumi Hamaguchi, Shunichi Hamaguchi, Yasuyuki Taooka, Takeshi Isobe, Yukari Tsubata

**Affiliations:** ^1^ Department of Internal Medicine, Division of Medical Oncology and Respiratory Medicine, Faculty of Medicine, Shimane University, Shimane, Japan; ^2^ Department of Respiratory and Critical Care Medicine, General Hospital of Ningxia Medical University, Yinchuan, China; ^3^ Department of Pathology, Shimane University Faculty of Medicine, Shimane University, Shimane, Japan; ^4^ Department of Respiratory Medicine, Second Affiliated Hospital of Ningxia Medical University, Yinchuan, China; ^5^ Division of Internal Medicine, Department of Respiratory Medicine, Medical Corporation JR Hiroshima Hospital, Hiroshima, Japan

**Keywords:** cyclooxygenase-2, single nucleotide polymorphism, promoter region, lung cancer risk, squamous cell carcinoma, Japanese

## Abstract

The single nucleotide polymorphisms of COX-2 gene, also known as *PTGS2*, which encodes a pro-inflammatory factor cyclooxygenase-2, alter the risk of developing multiple tumors, but these findings are not consistent for lung cancer. We previously reported that the homozygous COX-2 –1195A genotype is associated with an increased risk for chronic obstructive pulmonary disease (COPD) in Japanese individuals. COPD is a significant risk factor for lung cancer due to genetic susceptibility to cigarette smoke. In this study, we investigated the association between COX-2 –1195G/A polymorphism and lung cancer susceptibility in the Japanese population. We evaluated the genotype distribution of COX-2 –1195G/A using a polymerase chain reaction-restriction fragment length polymorphism assay for 330 newly diagnosed patients with lung cancer and 162 healthy controls. Our results show that no relationship exists between the COX-2 –1195G/A polymorphism and the risk of developing lung cancer. However, compared to the control group, the homozygous COX-2 –1195A genotype increased the risk for lung squamous cell carcinoma (odds ratio = 2.902; 95% confidence interval, 1.171–7.195; *p* = 0.021), whereas no association is observed with the risk for adenocarcinoma. In addition, Kaplan-Meier analysis shows that the genotype distribution of homozygous COX-2 –1195A does not correlate with the overall survival of patients with lung squamous cell carcinoma. Thus, we conclude that the homozygous COX-2 –1195A genotype confers an increased risk for lung squamous cell carcinoma in Japanese individuals and could be used as a predictive factor for early detection of lung squamous cell carcinoma.

## 1 Introduction

Lung cancer has the highest morbidity and mortality among cancers worldwide (Bray et al., 2018). There are many environmental risk factors for lung cancer and the most common is tobacco smoking (de Groot et al., 2018). However, exposure to environmental risk factors and genetic susceptibility are necessary for the development of lung carcinogenesis (Barta et al., 2019). Therefore, identifying genetic variants is vital for early prevention and screening of lung cancer.

Chronic inflammation plays an important role in carcinogenesis. Airway injuries related to inflammation caused by tobacco smoke or other environmental exposures increase the risk of developing lung cancer (Hecht, 2008). Moreover, inflammation-related genetic variants are highly associated with carcinogenesis for various cancers, including lung cancer (Rudnicka et al., 2019; Tan et al., 2019; Xiong et al., 2019).

The expression level of cyclooxygenase-2, a pro-inflammatory factor encoded by the COX-2 gene, is negligible in normal cells (Gurram et al., 2018). However, it is commonly overexpressed in various types of cancers and is implicated in the tumorigenesis, proliferation, metastasis, prognosis, and treatment of cancers (Tomozawa et al., 2000; Liu et al., 2001; Patti et al., 2002; Li et al., 2020). COX-2 is also involved in the molecular pathogenesis of chronic lung diseases (Park and Christman, 2006). Polymorphisms in the COX-2 gene alter the risk for chronic lung disease (Szczeklik et al., 2004; Xaubet et al., 2010). Three single nucleotide polymorphisms (SNPs) of the potential function, –1290G/A (rs689465), –1195G/A (rs689466), and –765G/C (rs20417), were identified in the COX-2 gene in esophageal cancer (Zhang et al., 2005). We previously reported that the homozygous COX-2 –1195A genotype is associated with an increased risk for chronic obstructive pulmonary disease (COPD) in the Japanese population (Chen et al., 2013). COPD is the single risk factor identified for the development of lung cancer after smoking exposure (Young et al., 2009). Chronic inflammation increases the risk for lung cancer by 2- to 3- fold in patients with COPD (Koshiol et al., 2009; Schwartz et al., 2016). Meta-analysis suggests that the emphysema detected visually on chest computed tomography (CT) and reduced forced expiratory volume in 1 s (FEV1) have strong effect on the increased odds of developing lung cancer (Wasswa-Kintu et al., 2005; Smith et al., 2012). Genetic analysis suggests that the genetic risk factors predisposing smokers to COPD and lung cancer may overlap (Young and Hopkins, 2011), and the key inflammatory-related genes and pathways impact the risk for lung cancer in a COPD-dependent manner (Watza et al., 2020). COX-2 is reported to be one of the candidate susceptibility genes related to inflammation involved in both COPD and lung cancer (Sekine et al., 2012).

The COX-2 –1195G/A gene polymorphism is functional and associated with an increased risk for various human cancers; however, the results are controversial in lung cancer (Zhang et al., 2005; Dong et al., 2010; Coskunpinar et al., 2011; Tang et al., 2011; Moraes et al., 2017). Therefore, this case-controlled study aimed to investigate the association between the COX-2 –1195G/A gene polymorphism and lung cancer susceptibility in the Japanese population.

## 2 Materials and Methods

### 2.1 Study Design and Participants

This study included 492 participants from the Japanese population. The enrolled 330 patients with lung cancer were newly diagnosed at the Shimane University Hospital or Higashi Hiroshima Medical Center between 2009 and 2012. The lung cancer cases consisted of 221 patients with lung adenocarcinoma, 85 patients with lung squamous cell carcinoma, 9 patients with small cell lung cancer, and 15 patients with the other types. The 162 healthy controls were randomly selected from participants who received an annual health screening at the Shimane Institute of Health Science between 2009 and 2012. Those who were diagnosed with any cancer or any respiratory disease should be excluded from the controls. Ethical approval was obtained from the Institutional Review Board at the Shimane University Faculty of Medicine and the Higashi Hiroshima Medical Center (approved number 1022). Each enrolled participant signed an informed consent form.

### 2.2 DNA Preparation and Genotype Determination

DNA from enrolled participants was isolated from whole blood. A polymerase chain reaction-restriction fragment length polymorphism (PCR-RFLP) assay was used for COX-2 polymorphism determination. The PCR reactions were performed in a reaction mixture system volume of 50 μl that contained 2.5 U Taq and 1 μl template DNA at a concentration of 50–150 ng/ml. The genotype of COX-2 –1195G/A was determined using the following specific primers: 5′- CCC TGA GCA CTA CCC ATG AT -3′ (forward) and 5′- GCC CTT CAT AGG AGA TAC TGG -3′ (reverse). The PCR cycling program was as follows: incubation at 96°C for 5 min followed by 32 cycles of 96°C for 20 s, 52°C for 30 s, and 72°C for 30 s with a final extension at 72°C for 6 min. The COX-2 273 bp PCR product was digested into 220 bp and 53 bp fragments with PvuII for the –1195G allele (restriction products: AA, 273 bp; GG, 220 bp + 53 bp; GA, 220 bp + 53 bp + 273 bp). The digested products were observed in a 2% agarose gel stained with ethidium bromide, and images were obtained under ultraviolet light.

### 2.3 Statistical Analysis

Data are presented as the number (%) of participants. All statistical analyses were performed using SPSS Statistics version 27.0 (IBM, Armonk, NY, United States). Demographic characteristics were analyzed using a Mann-Whitney *U*-test or a Chi-squared test. The distribution of genotypes was assessed using the Hardy-Weinberg equilibrium. Differences in the genotype distribution of COX-2 gene were analyzed using a Chi-squared test. The association between the genotype distribution of COX-2 –1195G/A and lung cancer risk was estimated using odds ratios (ORs) and a 95% confidence interval (95% CI) that were computed using logistic regression analysis. Survival distributions were estimated using Kaplan-Meier analysis and compared using the log-rank test. *P* < 0.05 was considered statistically significant.

## 3 Results

The demographic characteristics of all study participants are summarized in Table 1, which includes age, sex, and smoking history. We observed the lung cancer group had a higher median age than that of the control group (*p* < 0.001). Moreover, there was a higher prevalence of men and smoking in the lung cancer group than in the control group (all values of *p* < 0.001). The clinicopathology and the disease stages for the patients with lung cancer are listed in Table 2. Adenocarcinoma, squamous cell carcinoma, small cell carcinoma, and the others represented 67, 25.8, 2.7, and 4.5% of all patients with lung cancer, respectively. Stage I + II, stage III, and stage IV represented 3.9, 35.1, and 60.9% of all patients with lung cancer, respectively. The 330 patients with lung cancer and 162 healthy controls were genotyped for the COX-2 –1195G/A polymorphism (Table 3). The genotype distribution in the control group was consistent with the Hardy-Weinberg equilibrium. Moreover, for the genotype distribution of COX-2 –1195G/A, there is no significant difference between lung cancer patients and the controls (*p* = 0.17), and also no significant difference was obsearved between the adenocarcinoma group and the squamous cell carcinoma group (*p* = 0.158).

**TABLE 1:** Demographic characteristics of the study participants.

	Lung cancer (n = 330)	Control (n = 162)	*p*-value
Age			
Median	71	51	
Range	35–96	18–86	< 0.001
Sex			
Male (%)	255 (77.3)	99 (61.1)
Female (%)	75 (22.7)	63 (38.9)	< 0.001
Smoker			
No (%)	82 (24.8)	85 (52.5)	
Yes (%)	248 (75.2)	77 (47.5)	< 0.001

Note: *p*-values are presented for the comparison between the lung cancer group and the control group.

**TABLE 2:** Baseline clinicopathology and stage characteristics for patients with lung cancer.

	Number (%)
Histology	
Adenocarcinoma	221 (67.0)
Squamous cell carcinoma	85 (25.8)
Small cell carcinoma	9 (2.7)
Others	15 (4.5)
Stage	
I	9 (2.7)
II	4 (1.2)
IIIA	38 (11.5)
IIIB	78 (23.6)
IV	201 (60.9)

Note: values represent the number (%) of participants.

**TABLE 3:** The genotype distribution of COX-2 –1195G/A in patients with lung cancer and control participants.

	Lung cancer (n = 330)	Control (n = 162)	Unadjusted OR (95% CI)	*p*-value	Adjusted OR (95% CI)	*p*-value
Overall lung cancer						
Homozygous G	52 (15.8)	28 (17.3)	1		1	
Heterozygous G/A	167 (50.6)	93 (57.4)	0.967 (0.572–1.634)	0.9	0.949 (0.469–1.921)	0.884
Homozygous A	111 (33.6)	41 (25.3)	1.458 (0.814–2.610)	0.2	1.316 (0.608–2.845)	0.486

Note: OR: odds ratio; 95% CI: 95% confidence interval. The model is adjusted for the distributions of age, sex and smoking status in all the participants.

## 4 Outcome

**FIGURE 1:**
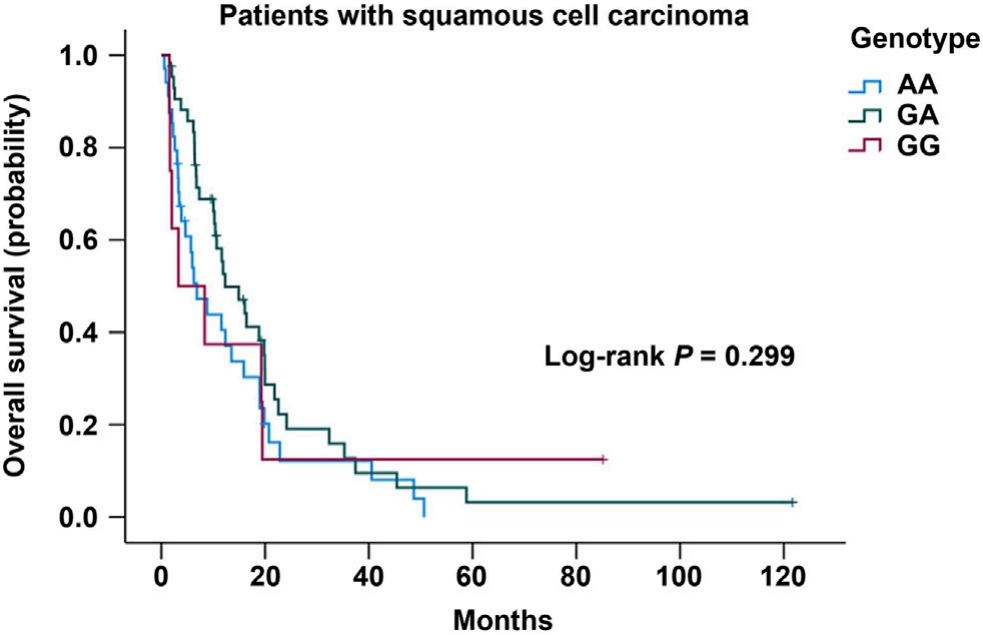
Kaplan-Meier analysis of overall survival in patients with lung squamous cell carcinoma stratified by the genotype of COX-2 –1195G/A.

No association on the risk of developing lung cancer with the genotype distribution of COX-2 –1195G/A was observed between the lung cancer and the control groups in both unadjusted analyses and adjusted analyses with age, sex, and smoking status (Table 3). Even though stratified by smoking status or sex in this study, we did not observe any association on the genotype distribution of COX-2 –1195G/A with the risk for lung cancer in both unadjusted and adjusted analyses with their respective factors (Table 4 and Table 5). In addition, no significant difference of the genotype distribution of COX-2 –1195G/A was found among the disease stages and the controls (Table 6). In the subgroup analyses, no increased risk was obsearved on the genotype distribution of the homozygous –1195A compared to the homozygous –1195G in the patients with stage IV (OR = 0.664; 95% CI, 0.346–1.272; *p* = 0.271). However, the multinomial logistic regression analysis represented that the genotype distribution of COX-2 –1195G/A was associated with the increasing risk for squamous cell carcinoma while not associated with adenocarcinoma (Table 7). The homozygous –1195A genotype increased the risk of 2.902 times for squamous cell carcinoma than the homozygous –1195G genotype (OR = 2.902; 95% CI, 1.171–7.195; *p* = 0.021). While no significant difference was observed from the heterozygous –1195G/A genotype for developing squamous cell carcinoma (OR = 1.618; 95% CI, 0.681–3.843; *p* = 0.275). To assess whether the prognosis was affected by the genotype of COX-2 –1195G/A, we compared the overall survival (OS). The median OS did not correlate with the genotype distribution of COX-2 –1195G/A among patients with squamous cell carcinoma (log-rank: *p* = 0.299) (Figure 1). In addition, the clinicopathology and sex were not related to the OS in patients with lung cancer (Supplementary Figure S1 and Supplementary Figure S2). Furthermore, there is no significant difference in the OS between the genotype of COX-2 –1195G/A stratified by sex (Supplementary Figure S3 and Supplementary Figure S4). Based on the above outcome, we summarized that the homozygous COX-2 –1195A genotype might increase the risk for lung squamous cell carcinoma in the Japanese population but no effect on the prognosis of squamous cell carcinoma.

**TABLE 4:** The genotype distribution of COX-2 –1195G/A gene stratified by smoking status.

	Lung cancer (n = 330)	Control (n = 162)	Unadjusted OR (95% CI)	*p*-value	Adjusted OR (95% CI)	*p*-value
Non-smokers						
Homozygous G	18 (22.0)	18 (21.2)	1		1	
Heterozygous G/A	45 (54.9)	45 (52.9)	1.000 (0.462–2.166)	1.0	0.975 (0.346–2.749)	0.962
Homozygous A	19 (23.1)	22 (25.9)	0.864 (0.352–2.117)	0.749	1.039 (0.329–3.279)	0.948
Smokers						
Homozygous G	34 (13.7)	10 (13.0)	1		1	
Heterozygous G/A	122 (49.2)	48 (62.3)	0.748 (0.343–1.631)	0.465	0.983 (0.373–2.590)	0.972
Homozygous A	92 (37.1)	19 (24.7)	1.424 (0.602–3.368)	0.421	1.522 (0.533–4.343)	0.433

Note: OR: odds ratio; 95% CI: 95% confidence interval. The model is adjusted for the distributions of age and sex in the respective participants.

**TABLE 5:** The genotype distribution of COX-2 –1195G/A gene stratified by sex.

	Lung cancer (n = 330)	Control (n = 162)	Unadjusted OR (95% CI)	*p*-value	Adjusted OR (95% CI)	*p*-value
Male						
Homozygous G	40 (15.7)	18 (18.2)	1		1	
Heterozygous G/A	122 (47.8)	56 (56.6)	0.980 0.517–1.859)	0.952	1.253 (0.526–2.985)	0.611
Homozygous A	93 (36.5)	25 (25.3)	1.674 (0.823–3.406)	0.155	1.802 (0.707–4.588)	0.217
Female						
Homozygous G	12 (16.0)	10 (15.9)	1		1	
Heterozygous G/A	45 (60.0)	37 (58.7)	1.014 (0.394–2.608)	0.978	0.620 (0.181–2.127)	0.447
Homozygous A	18 (24.0)	16 (25.4)	0.938 (0.320–2.750)	0.906	0.676 (0.171–2.669)	0.576

Note: OR: odds ratio; 95% CI: 95% confidence interval. The model is adjusted for the distributions of age and smoking status in the respective participants.

**TABLE 6:** The comparison on the genotype distribution of COX-2 –1195G/A gene among lung cancer patients with different disease stages and control participants.

	Stage IV (n = 201)	Stage IIIB (n = 78)	Stage IIIA (n = 38)	Stage I + II (n = 13)	Control (n = 162)	*p*-value
Homozygous G	29 (14.4)	17 (21.8)	4 (10.5)	2 (15.4)	28 (17.3)	
Heterozygous G/A	108 (53.7)	35 (44.9)	19 (50.0)	5 (38.5)	93 (57.4)	0.371
Homozygous A	64 (31.8)	26 (33.3)	15 (39.5)	6 (46.2)	41 (25.3)	

Note: *p*-values are presented for comparison among lung cancer patients with different disease stages and control participants.

**TABLE 7:** The genotype distribution of COX-2 –1195G/A in adenocarcinoma and squamous cell carcinoma.

	Lung cancer (n = 330)	Control (n = 162)	OR (95%CI)	*p*-value
Adenocarcinoma				
Homozygous G	39 (17.6)	28 (17.3)	1	
Heterozygous G/A	110 (49.8)	93 (57.4)	0.849 (0.486–1.484)	0.566
Homozygous A	72 (32.6)	41 (25.3)	1.261 (0.679–2.341)	0.463
Squamous cell carcinoma				
Homozygous G	8 (9.4)	28 (17.3)	1	
Heterozygous G/A	43 (50.6)	93 (57.4)	1.618 (0.681–3.843)	0.275
Homozygous A	34 (40.0)	41 (25.3)	2.902 (1.171–7.195)	0.021

Note: OR: odds ratio; 95% CI: 95% confidence interval.

## 5 Discussion

This study analyzed the association between genotypes of the COX-2 –1195G/A polymorphism and different clinicopathology of lung cancer, and the results demonstrate that the homozygous COX-2 –1195A genotype was associated with the increased risk of developing lung squamous cell carcinoma. To date, the related studies have inconsistent results. The homozygous COX-2 –1195A genotype increased the risk of lung cancer development in the Turkish population, wherein patients with lung squamous cell carcinoma represented 53.2% (Coskunpinar et al., 2011). By contrast, a study from the Brazilian population concluded that the COX-2 –1195G/A polymorphism was not associated with the risk for lung cancer (Moraes et al., 2017), which is in agreement with the results from our present study. On the other hand, the number of patients diagnosed with lung squamous cell carcinoma observed in the Brazilian study is 39.4% (Moraes et al., 2017), and that of our study is 25.8%. Based on these results, we hypothesized that the discrepancy observed in the number of patients with homozygous COX-2 –1195A might be due to the different distribution of lung cancer clinicopathology. Indeed, our results reveal that the homozygous COX-2 –1195A genotype increases the risk for lung squamous cell carcinoma.

Increased levels of COX-2 expression were observed in bronchial precursors of squamous cell carcinoma using immunohistochemistry (Petkova et al., 2004; Mascaux et al., 2005). The substitution of –1195G > A creates a binding site for a transcription factor c-MYB in the COX-2 promoter region, which regulates the balance among cell division, differentiation, and survival, resulting in facilitation of COX-2 transcription (Ramsay et al., 2003; Zhang et al., 2005). Moreover, the homozygous COX-2 –1195A genotype exhibit a significant increase in the mRNA level of COX-2 expression than the genotypes of homozygous –1195G and/or the heterozygous –1195 GA in esophageal tissue (Zhang et al., 2005). In lung cancer, a Brazilian study demonstrated that the homozygous –1195A did not increase the mRNA expression of COX-2 compared to the other genotypes (Moraes et al., 2017). However, the 34 lung tumor specimens comprised 19 (55.9%) adenocarcinoma cases and 15 (44.1%) squamous cell carcinoma cases in the Brazilian study. The association between COX-2 -1195G/A polymorphism and the risk for lung cancer may be pathologically and ethnically dependent. Further studies with larger sample sizes that include populations of different races and analyses stratified by histology classifications are necessary to investigate the controversial results.

**FIGURE 2:**
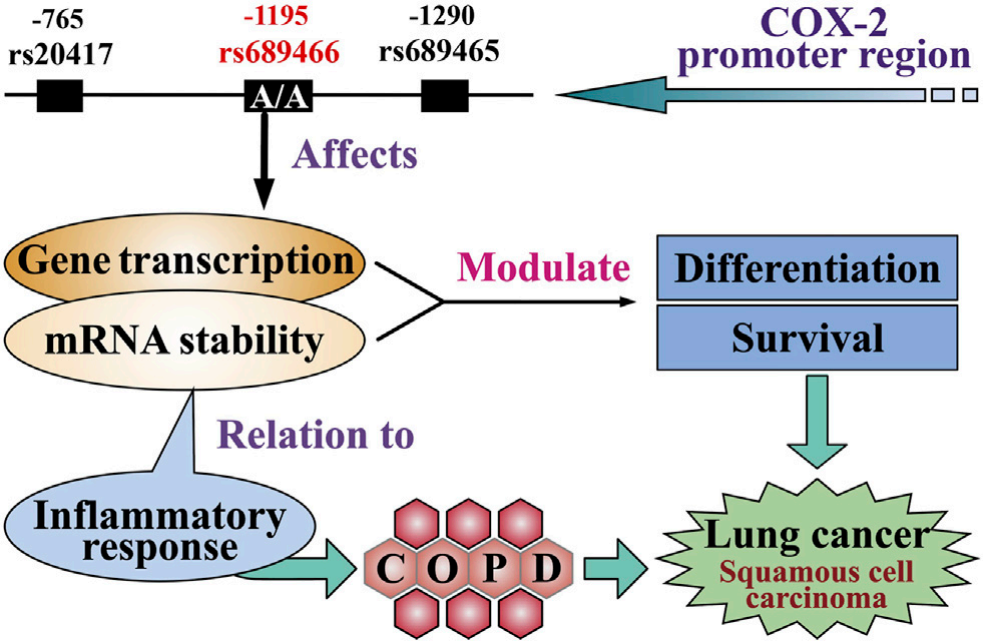
Potential function of the COX-2 single nucleotide polymorphisms in lung cancer. The genotype of homozygous COX-2 –1195A in the COX-2 promoter region affects gene transcription, thereby increasing the expression of COX-2 in lung cells. The inflammatory response is related to mRNA stability. Both factors enhance the level of differentiation of lung cells and promote the development of lung squamous cell carcinoma. The inflammatory response also leads to chronic obstructive pulmonary disease and lung cancer.

We previously demonstrated that the homozygous COX-2 –1195A genotype increased the risk for COPD in Japanese individuals (Chen et al., 2013). A Swedish study showed that the association with a lower FEV1 was higher for patients with lung squamous cell carcinoma than those with lung adenocarcinoma (Purdue et al., 2007). Further, the presence of emphysema, a typical manifestation of COPD on a chest CT scan, is associated with significantly increased odds of developing squamous cell carcinoma (Wang et al., 2018). Moreover, smoking is a major risk factor in the pathogenesis of lung squamous cell carcinoma and COPD, and it upregulates inflammation-related genes, including COX-2, in tracheal smooth muscle cells (Yang et al., 2009). Therefore, a potential link might exist between the functional COX-2 SNPs, COPD, and lung cancer, particularly for lung squamous cell carcinoma (Figure 2) (Lee et al., 2009). Considering the possible relationship between pulmonary function, emphysema CT scan parameters, smoking status, and COX-2 –1195A homozygosity, further studies are required.

We did not observe any association between the risk for lung cancer and the COX-2 –1195G/A polymorphism stratified by smoking status. By contrast, a previous study from the Taiwanese population reported that the enrolled patients who smoked and carried the A allele of rs2066826 in the COX-2 intron 6 had an increased risk of 2.21 for lung cancer. (Liu et al., 2010). Further studies are needed to comprehensively analyze the functional COX-2 polymorphisms in addition to geographic populations.

The relationship between COX-2, COPD, and lung cancer is complicated. Epithelial-to-mesenchymal transition (EMT) is critical for lung carcinogenesis and observation of a malignant phenotype, and inhibition of COX-2 reverses EMT-induced changes in lung cancer patients (Dohadwala et al., 2006; Peebles et al., 2007). EMT in COPD and the resultant association with the risk for lung cancer have not been completely elucidated. Fundamental research is necessary to identify the molecular mechanisms linking these diseases.

Genotyping patients and identifying those with homozygous COX-2 –1195A could be combined with identifying emphysema using chest CT scans to serve as predictive markers for the early prevention and screening of lung squamous cell carcinoma. The increased odds of developing lung cancer in the presence of emphysema on CT may prove to be useful in targeting resources for the prevention and screening of lung squamous cell carcinoma. In addition, our findings suggest that either shared host susceptibility or an uncharacterized novel mechanism promotes the pathogenesis of both COPD and lung squamous cell carcinoma. It is necessary to further explore the benefit of clinical interventions to prevent or detect lung cancer after a patient is diagnosed with emphysema.

On the other hand, it has been reported that high levels of COX-2 mRNA transcription are associated with a more aggressive phenotype and poor prognosis for patients with non-small cell lung cancer (NSCLC) (Brabender et al., 2002). The homozygous COX-2 –1195A genotype is associated with poor overall survival in Chinese patients with NSCLC treated with chemoradiotherapy or radiotherapy alone (Bi et al., 2010). Although the homozygous COX-2 –1195A increased the risk for lung squamous cell carcinoma, this genotype did not correlate with poor prognosis in our study when evaluated using median overall survival. One reason for this discrepancy might be the fact that certain genetic markers are ethnicity-specific; another reason might be that different treatment regimens play a role in the prognosis of lung cancer and influence the effects of the COX-2 genotypes.

The three limitations of this study are listed as follows: the number of enrolled participants was low; only patients from the Japanese population were included; it was an imbalance of the baseline characteristics between the patients with lung cancer and the control participants.

In conclusion, the homozygous COX-2 –1195A increased the risk of developing lung squamous cell carcinoma and might be used as a predictive marker for early detection and screening of lung squamous cell carcinoma in Japanese individuals, but not as a predictive marker for the prognosis.

## Data Availability

The data analyzed in this study is subject to the following licenses/restrictions: The data that support the findings of this study are available from Shimane university hospital but restrictions apply to the availability of these data, which were used under license for the current study, and so are not publicly available. Requests to access these datasets should be directed to Yukari Tsubata, ytsubata@med.shimane-u.ac.jp.
