# Supplementary material for: The Association Between Cyclooxygenase-2 –1195G/A (rs689466) Gene Polymorphism and the Clinicopathology of Lung Cancer in the Japanese Population: A Case-Controlled Study
Source: Front Genet. 2022 Apr 5;13:796444. doi: 10.3389/fgene.2022.796444 (PMC9016323; doi:10.3389/fgene.2022.796444)
Supplement: Supplementary file 1 [file DataSheet1.docx]

**Supplementary Figures**


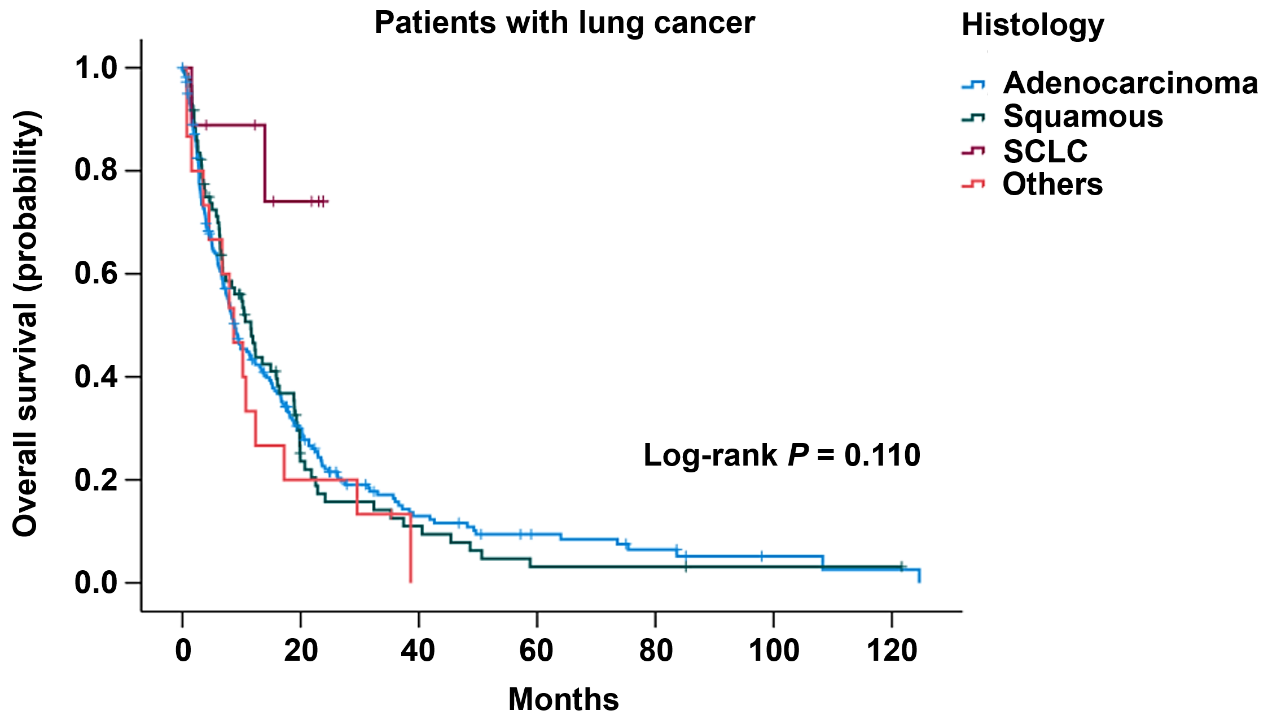


**Supplementary Figure S1** Kaplan-Meier analysis of overall survival in patients with lung cancer stratified by histology classifications.


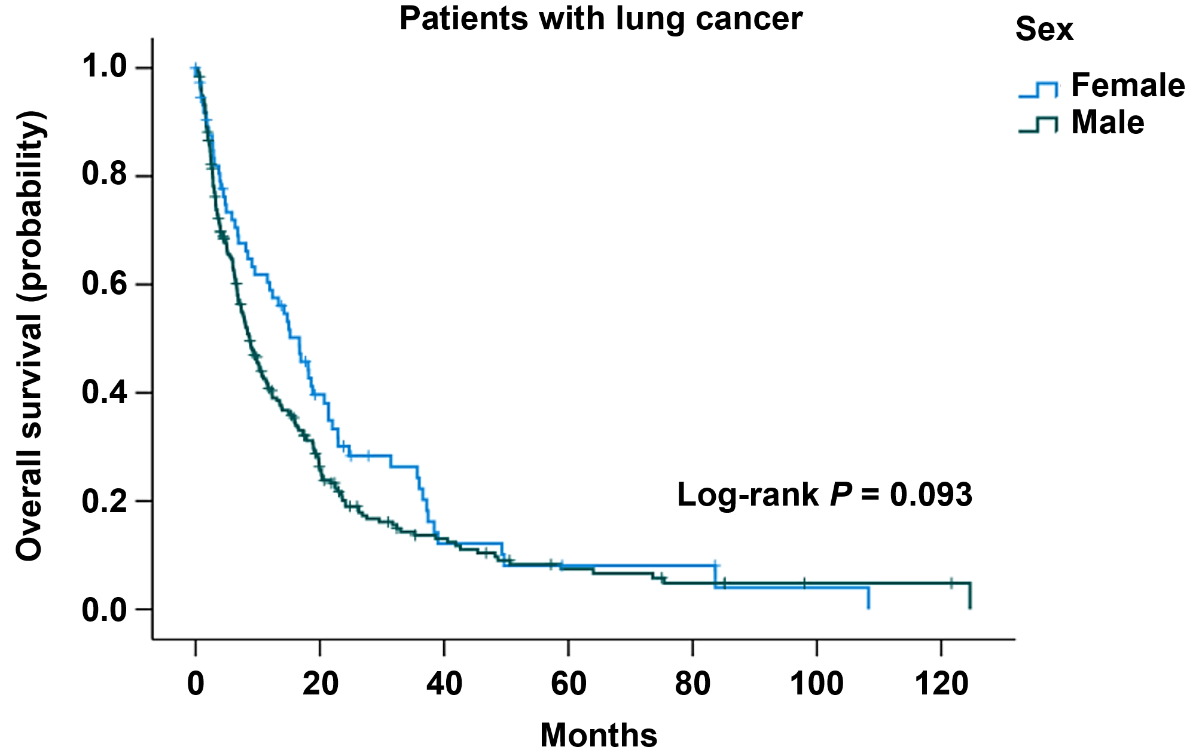


**Supplementary Figure S2** Kaplan-Meier analysis of overall survival in patients with lung cancer stratified by sex.


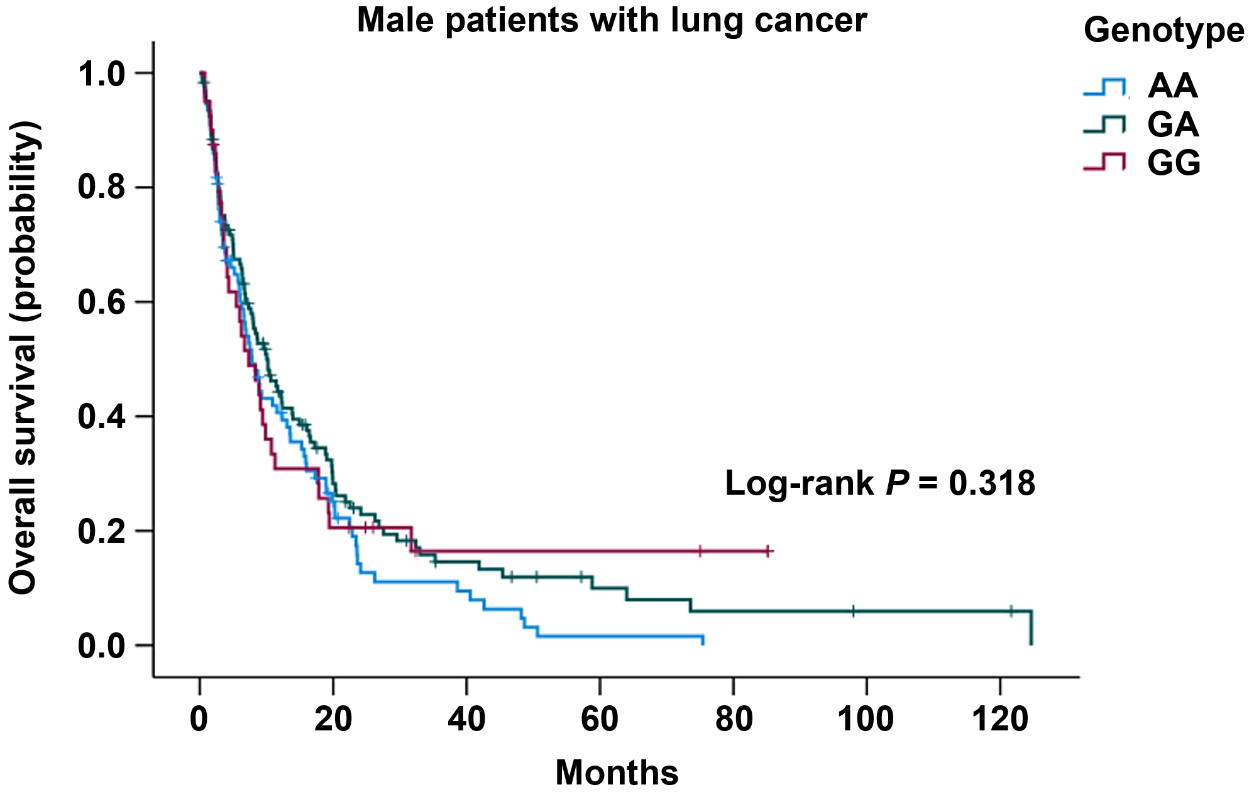


**Supplementary Figure S3** Kaplan-Meier analysis of overall survival in male patients with lung cancer stratified by the genotype of COX-2 --1195G/A.


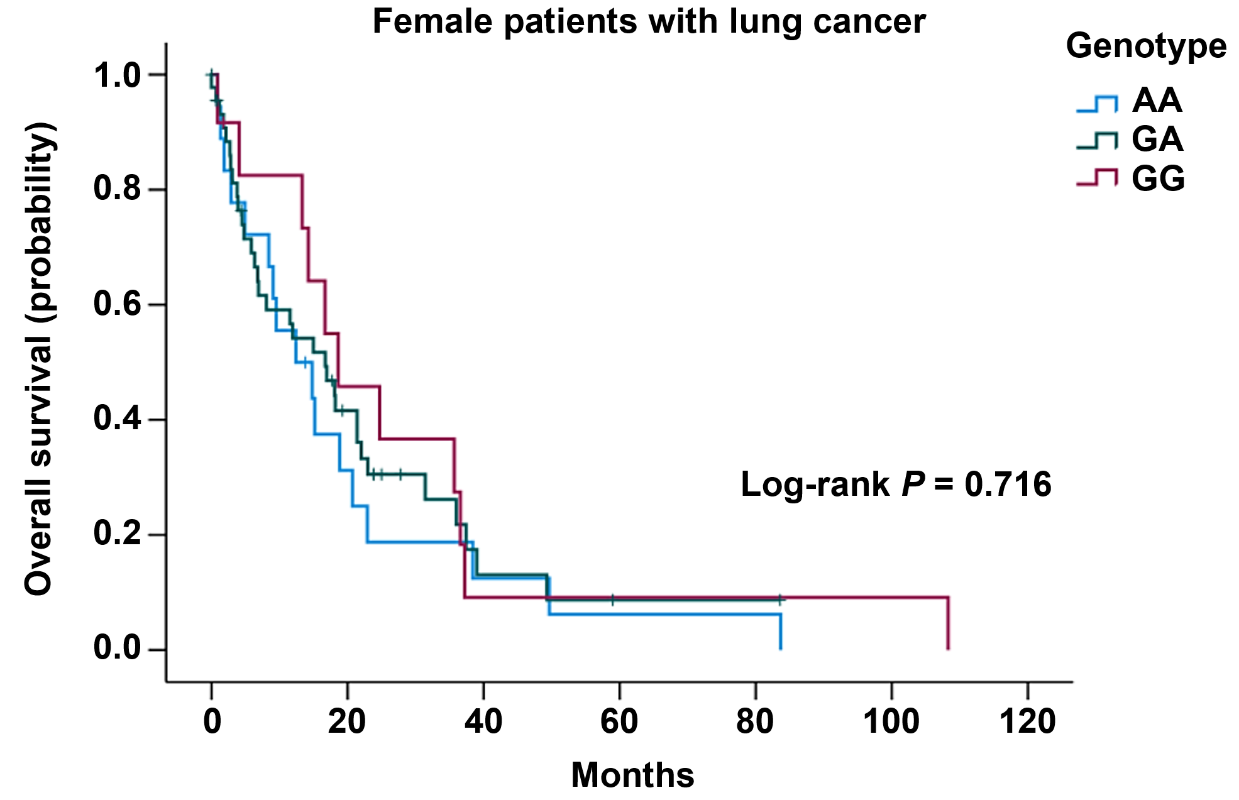


**Supplementary Figure S4** Kaplan-Meier analysis of overall survival in female patients with lung cancer stratified by the genotype of COX-2 --1195G/A.
